# Neural dynamic foundations of a theory of higher cognition: the case of grounding nested phrases

**DOI:** 10.1007/s11571-023-10007-7

**Published:** 2023-10-04

**Authors:** Daniel Sabinasz, Mathis Richter, Gregor Schöner

**Affiliations:** 1 Institute for Neural Computation, Ruhr-University Bochum, Bochum, Germany; 2 Neuromorphic Computing Lab, Intel Germany GmbH, Feldkirchen, Germany

**Keywords:** Neural process model, Embodied cognition, Higher cognition, Language grounding, Dynamic field theory, Concepts, Conceptual structure

## Abstract

Because cognitive competences emerge in evolution and development from the sensory-motor domain, we seek a neural process account for higher cognition in which all representations are necessarily grounded in perception and action. The challenge is to understand how hallmarks of higher cognition, productivity, systematicity, and compositionality, may emerge from such a bottom-up approach. To address this challenge, we present key ideas from Dynamic Field Theory which postulates that neural populations are organized by recurrent connectivity to create stable localist representations. Dynamic instabilities enable the autonomous generation of sequences of mental states. The capacity to apply neural circuitry across broad sets of inputs that emulates the function call postulated in symbolic computation emerges through coordinate transforms implemented in neural gain fields. We show how binding localist neural representations through a shared index dimension enables conceptual structure, in which the interdependence among components of a representation is flexibly expressed. We demonstrate these principles in a neural dynamic architecture that represents and perceptually grounds nested relational and action phrases. Sequences of neural processing steps are generated autonomously to attentionally select the referenced objects and events in a manner that is sensitive to their interdependencies. This solves the problem of 2 and the massive binding problem in expressions such as “the small tree that is to the left of the lake which is to the left of the large tree”. We extend earlier work by incorporating new types of grammatical constructions and a larger vocabulary. We discuss the DFT framework relative to other neural process accounts of higher cognition and assess the scope and challenges of such neural theories.

## Introduction

How humans achieve higher cognition continues to fascinate cognitive scientists, neuroscientists, psychologists, computer scientists, and other scholars with an interest in the human condition. The neural basis for mental capacities such as using language to generate and understand narratives, thinking to reason, plan, or solve problems, using analogy to transfer knowledge to new domains, remains largely unknown. One hallmark of higher cognition is that it seems to abstract from the concrete sensory or motor manifestations of the objects or events that cognition is about. That abstraction is captured when higher cognition is described as a form of symbol manipulation (Newell and Simon 1972). The symbols are the abstract representations of objects or events, and their flexible manipulation captures productivity, the capacity to generate ever new chains of thoughts or actions, and compositionality, the capacity to create new thoughts or ideas from a given set of symbols (Fodor and Pylyshyn 1988). Systematicity describes the hypothesis that the way symbols are used and combined is constrained by certain patterns of regularity, described as rules.

Cognition as symbol manipulation aligns with the notion of (digital) computation. Computers operate in a rule-based way on internal states that can be thought of as physical instantiations of symbols. Algorithms systematically organize sequences of such operations to produce an output. How such algorithms may describe or emulate human thought has been a major topic of research on cognitive architectures of the human mind (e.g., Laird 2019; Anderson 2013).

Clearly, the human brain does not look like a digital computer. There is no obvious way how neural states are stored and accessed by a central processor, how algorithms are stored or implemented in the brain. Nor is there an obvious way how neural states come to stand for the objects or events that the algorithm is making computations about. So the computer metaphor, and, more generally, the notion of cognition as symbol manipulation, do not by themselves explain how the human brain achieves cognition.

There are two different ways in which researchers approach the challenge of understanding the neural foundations of human cognition. These mirror longstanding debates in cognitive linguistics between the view of language as an innate, special module of the human mind (Pinker 2003) versus a view of language as emerging from sensory-motor competences in development and evolution (Elman et al. 1997). This special issue will likely contain contributions aligned with either approach. The first approach seeks a general neural mechanism, a “neural Turing machine”, that enables neural networks to manipulate symbols productively and systematically (Zylberberg et al. 2013; beim Graben and Potthast 2014; Carmantini et al. 2017; Lake et al. 2017). This would be a neural *implementation* of symbol manipulation (Marr 1982; Fodor and Pylyshyn 1988). Linking symbols to their referent through sensory-motor processes (Harnad 1990; Barsalou 1999), is considered a separate problem in this view.

The other approach addresses the sensory-motor grounding of cognition head on and asks instead, how grounded processes may achieve the seeming flexibility and capacity for abstraction described by the notions of productivity, systematicity, and compositionality. This view postulates that higher cognitive competences *emerge* from the dynamics of the neural networks that are coupled to the world through the sensory-motor systems, consistent with the evolutionary (Tomasello 2014) and developmental (Thelen and Smith 1994; McClelland et al. 2010; Samuelson et al. 2011) primacy of sensory-motor behaviors. Empirical support comes from a range “embodiment effects” (Newen et al. 2018).

The goal of this paper is to propose a concrete theoretical framework to develop this second approach toward a neural account for higher cognition. Embodiment and grounding plays a central role in this approach. Because sensory-motor processes unfold in closed loop with the environment, their theoretical understanding invokes dynamical systems ideas including stability (Schöner 2008). Dynamic Field Theory (DFT; Schöner and Spencer 2015) extends this dynamical systems perspective to cognition postulating that cognitive processes inherit stability properties from the sensory-motor domain (Van Gelder 1998). The related notion of *neural dynamics* goes back at least to Stephen Grossberg’s pioneering work (Grossberg 1978), and is implied in connectionist modeling of recurrent neural networks (Usher and McClelland 2001). The DFT approach originated from work on the developmental foundation of cognition including accounts for perseverative reaching in infants (Thelen 2001), the development of working memory (Simmering et al. 2008; Johnson et al. 2014), visual categories (Perone and Spencer 2014), cognitive control (Buss and Spencer 2014), among many other forms of early cognition (Schöner and Spencer 2015).

In the classical conception of cognition as symbol manipulation, the capacity to generalize and operate at an abstract level of representation is formalized through the notion of mathematical functions. For instance, relations such as “to the left of” or “contained in” are framed as functions that take two arguments, the reference and the target object, and return a truth value. This makes explicit the abstraction and generality of these cognitive operations that depend only on the information passed to the functions, the objects’ locations, not on the sensory-motor details of the representation of each object. How would a neural dynamic account grounded in the sensory-motor domain provide this level of generality and abstraction? Such issues were debated early in the connectionist challenge to classical information processing, then around the question of how the past tense of verbs may be formed: How may rules be applied to a pseudo-word for which a suitable neural representation has not yet been built (Pinker 2006)? The radical variant of the proposed solution is implemented in deep convolutional neural networks: Pieces of neural circuitry are copied through weight sharing across an entire layer of the network. The operation encoded in this circuitry can be then applied anywhere in an image (Santoro et al. 2017). That solution is neither neurally plausible, nor does it scale reasonably. In DFT, coordinate transforms provide the solution (Richter et al. 2021) to this “neural pointer problem” (Ballard et al. 1997). Neural circuitry implementing the relation “to the left of”, for instance, may be specific to the reference object being positioned at its center. To apply that circuitry to a reference object anywhere in the visual array, the array is transformed into a coordinate frame centered on the candidate reference object via a steerable neural map (Deneve and Pouget 1998).

This paper continues the expansion of DFT as a neural account of higher cognition beyond initial forays into relational thinking (Lipinski et al. 2012; Richter et al. 2014), mental mapping (Kounatidou et al. 2018), and word learning (Bhat et al. 2022) (for review, see Schöner (2023)). The critical open question is, if and how DFT can provide a neural account for the flexibility of higher cognition described by productivity, systematicity, and compositionality. We will address this question around the exemplary problem of how *nested* phrases can be represented and perceptually grounded. Such phrases join “atomic” linguistic units into “molecular” linguistic units at several layers of recursion. We show how *structured representations* (Jackendoff 2002) may be neurally realized by flexibly binding separate neural representations of linguistic units through an index dimension in order to represent their interdependencies within the nested phrase. We show how the perceptual grounding of such structured representations is autonomously generated in sequences of neural processing steps that take these interdependencies into account.

In the next section, we review the neural principles of DFT including the three key neural mechanisms of binding, coordinate transformation, and sequence generation. Then we present a neural dynamic architecture that perceptually grounds nested phrases. For example, the sentence “the blue ball approaches the big tree, which is to the left of the lake and to the right of the house” is perceptually grounded by attentionally selecting the designated objects in a visual scene. We relegate the analysis of how this approach relates to alternative neural accounts of higher cognition to the Discussion, where we also point to limitations and future challenges.

## Dynamic field theory

Dynamic Field Theory (DFT; Schöner and Spencer 2015) is a theoretical framework to understand the neural basis of embodied cognition. The neural accounts provided within DFT are not primarily concerned with mapping processes onto specific brain regions, although such mappings are possible (Buss et al. 2021). Instead, DFT postulates a set of principles that capture constraints of the brain networks from which cognitive function emerges. Most of these principles are consistent with connectionism (Thomas and McClelland 2008), neuroconstructivism (Mareschal et al. 2007), or neural networks modeling in general, but some are more specific and thus more constraining than recognized in this broader literature. This first section reviews the principles of DFT.

### Neural dynamics, fields and peaks

Like most neural network models, DFT uses an activation concept to describe the state of neural networks by continuous variables. The spiking mechanism of real neurons is replaced by a sigmoidal threshold function. The resulting population level description (Schöner 2019) can be derived under some conditions as a mean field approximation of neural activity (Gerstner et al. 2014, Part 3).

Also shared with all neural network models is the postulate that the functional meaning of neural activation patterns derives exclusively from the connectivity to and from a given neural population. Neural populations cannot exchange “messages” or “call functions”, they are merely coupled to other neural populations. That pattern of connectivity ultimately links any neural population to sensory surfaces and to motor systems, so that neural representations within DFT always possess sensory-motor grounding (Barsalou 2008).

**Fig. 1:**
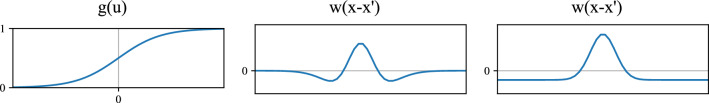
The sigmoid threshold function, $\sigma (u)$, and two typical forms of the interaction kernel, $w(x-x^\prime )$

Less universally shared is the assumption of DFT that neural activation, *u*(*t*), evolves continuously in time, *t*. Although many neural network models use discrete time steps at which the state of the network is updated, that is primarily a conceptual simplification rather than a principled position. Because neural spiking is a priori asynchronous across neurons, the discrete times of spiking events can best be viewed as a sampling of continuous time, not as clocked computation. Continuous time is also appropriate to understand the link of neural to sensory-motor processes and actual movement generation. Finally, thinking of neural activation as evolving in time is critical to understanding recurrent neural networks. In the mathematical formalization of *neural dynamics* (Grossberg 1978):

1 $$\begin{aligned} \tau \dot{u} = - u + h + \textrm{inputs}, \end{aligned}$$

the “$-u$” term is inherited from the dynamics of neural membranes (as in integrate-and-fire neural models (Gerstner et al. 2014)). This term creates stability: The fixed point, $u=h +\textrm{inputs}$ (for constant input), of this neural dynamics is an attractor to which any initial activation level converges on the time scale, $\tau \approx 10$ ms, inherited from membrane properties. In the absence of input, activation converges to the resting state, $h<0$. By convention, zero is chosen as the threshold for transmission of activation through a sigmoidal threshold function,

2 $$\begin{aligned} \sigma (u) = \frac{1}{1 + \exp (- \beta u)}, \end{aligned}$$

whose steepness is fixed by $\beta >0$ (Fig. 1). Input shifts the sub-threshold attractor, $h+\textrm{input}$, as long as this activation level remains below zero.

Stability is a critical property of all functional activation states in DFT, clearly relevant when activation patterns steer behavior in closed sensory-motor loops, but also critical when activation patterns drive purely mental processes. In particular, decisions must be maintained and stabilized against competing neural states. Endowing supra-threshold activation with stability requires recurrent neural connectivity that is organized to protect activation patterns from decay and from competing states. The mathematics of this are well understood in a localist picture in which neural activation variables are organized topologically. Using a continuous representation of the underlying topology, such neural activation variables form *neural fields*, *u*(*x*), defined over low-dimensional spaces, *x* (more on these spaces below). A neural dynamics supporting localized stable states is then (Amari 1977; Coombes 2005) (extensive review in Coombes et al. (2014))

3 $$\begin{aligned} \tau {\dot{u}}(x, t) = -u(x, t) + h + s(x, t) + \int w(x - x')\, g(u(x', t)) \, dx' + q\, \xi (x, t) \end{aligned}$$

where *s* denotes external input, and *w* is a pattern of recurrent connectivity that excites locally and inhibits globally across the field:

4 $$\begin{aligned} w(x-x') = - w_\textrm{inhib} + w_\textrm{exc}\exp \left( -\frac{(x-x')^2}{2\sigma _\textrm{kernel} }\right) \end{aligned}$$

(illustrated in Fig. 1, right). Similar patterns of neural connectivity are common in the brain (Jancke et al. 1999), often in the form illustrated in Fig. 1, middle (see chapter 7.5 of Dayan and Abbott (2001) for review). Neural fluctuations are modelled by additive Gaussian white noise, $\xi (x,t)$, of strength, *q*.

**Fig. 2:**
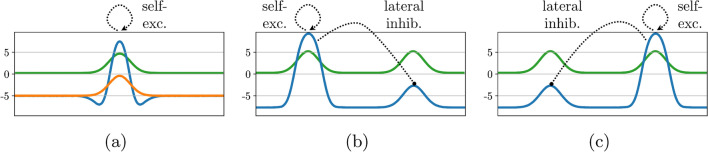
Detection and selection decisions enabled by recurrent connectivity (interaction) within neural dynamic fields. **a** Localized input (green) may induce a sub-threshold hill of activation (orange) or a supra-threshold peak of activation (blue) depending on the prior state of the field. **b** In response to bi-modal input (green), a single supra-threshold activation peak (blue) on the left is generated due to global inhibition. **c** Same as center panel, but the right-most location is selected, potentially due to prior activation or noise. (Color figure online)

The recurrent neural interaction stabilizes supra-threshold peaks of activation, the elementary forms of localist representations central to DFT (Fig. 2a). Such peaks may co-exist bi-stably with sub-threshold hills of activation for weak localized input patterns. A sub-threshold activation pattern loses stability for sufficiently strong localized input in the *detection instability*, leading to a switch to the supra-threshold peak of activation. Once such a peak has been created, it persists even when the inducing input is weakened, a form of self-stabilization of the detection decision. The peak decays only when localized input becomes so weak that local excitatory interaction is no longer sufficient to stabilize it in the reverse detection instability. For strong excitatory interaction or high levels of background activation (due to an elevated resting level or a homogeneous boost input, for instance), this reverse instability may not be reached even when localized input is removed entirely, leading to sustained activation, a standard model of working memory.

Inhibitory recurrent connectivity enables *selection* in which only one localized peak of supra-threshold activation is generated in response to input with multiple local maxima (Fig. 2b, c). Selection may be biased by input strength, but also the prior activation state, leading to the stabilization of selection decisions.

### Dimensions

What are these dimensions, *x*, in terms of which localized representations can be organized? Ultimately, the dimensions originate in the forward connectivity from a sensory surface to a neural field, or from a neural field to the motor system. Such forward projections enable feature extraction and movement parameter encoding, respectively. Neurons sampling the fields are effectively “tuned to” a set of feature dimensions that they “encode”. It is this forward connectivity that enables the sensory-motor grounding of cognitive processes.

DFT makes two specific postulates about the dimensions of these feature/parameter spaces. First, the number of dimensions represented by a given population is strongly limited, typically three to five. This comes from a scaling argument: As the number of feature dimensions increases, the number of neurons needed to sample a feature space increases combinatorially. Second, two dimensions are shared among many of the fields: a two-dimensional representation of visual space for perceptual representations or a corresponding representation of the hand’s movement direction in space for motor representations. This enables *binding through space* in which localized activation in one low-dimensional field can be linked to localized activation in another low-dimensional field by exciting a hyper-cylinder localized within the shared spatial dimensions and extending along the other dimensions (Schneegans et al. 2016, see Sect. 2.3).

How may representations of concepts or categories fit into this framework? Categorical representations may not carry along any feature dimensions, but may still be embedded in a space within which selection takes place. In DFT, *neural nodes* provide the substrate for such categorical representations. These are neural activation variables, *u*(*t*), whose dynamics

5 $$\begin{aligned} \tau {\dot{u}}(t) = -u(t) + h + s(t) + w_{\text {se}}\, g(u(t)) - \textrm{competition} + w_{\xi }\, \xi (t) \end{aligned}$$

is analogous to that of fields. Excitatory interaction takes the form of self-excitation with strength $w_\text {se}$. This enables the detection and reverse detection instabilities, endowing nodes with the fundamental bistability between an *on*-state (output close to 1) and *off*-state (output close to 0). Selection results from reciprocal inhibitory coupling to other neural nodes.

### Architectures

**Fig. 3:**
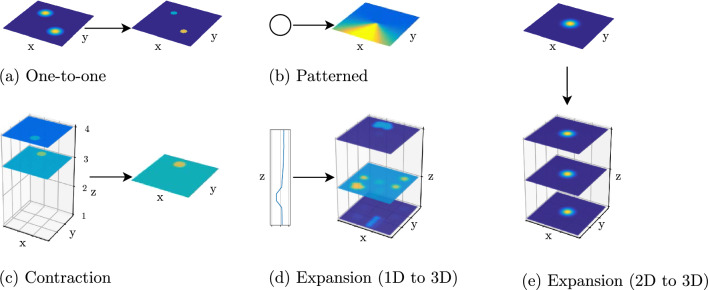
Different forms of coupling between neural fields

The output of a field or node may provide input to other fields or nodes, and receive input from those other fields or nodes (Fig. 3). Such coupling can bring about different kinds of mappings. In *one-to-one coupling* (a), the dimensions and their meaning are preserved. *Contraction coupling* (c) reduces the dimensionality by summing over one or more dimensions. *Expansion coupling* (d, e) increases the dimensionality by providing input that is constant along the extra dimensions (“ridge” or “slice” input). In this way, nodes may provide homogeneous boosts across all dimensions of a field, in effect controlling the resting level of the field. Finally, *patterned coupling* (b) from a node to a field may pre-activate particular regions in the field.

Neural dynamic architectures are built by coupling fields and nodes. The capacity of a field to make detection and selections decisions, or to build working memories, is realized by its dynamic regime, which attractors and which instabilities may occur as input is varied. As long as it is sufficiently weak, coupling preserves the dynamic regime of a field, a key property of DFT. The different forms of coupling illustrated above also preserve the meaning of the field dimensions.

### Sequences

Central to DFT is the postulate that functionally significant states are attractors of the neural dynamics. This raises the question of how the sequences of states may emerge that underlie sequential cognitive processing or motor acts (Sandamirskaya and Schöner 2010; Sandamirskaya 2016; Tekülve et al. 2019). DFT addresses this question in two steps. First, to terminate any current activation state, the corresponding attractor must be made unstable, ultimately leading to the decay of the underlying activation peak in a reverse detection instability. Second, as the current activation state decays, the system may move to a new activation state that may have already existed as an attractor (through multistability) or that becomes stable as inhibition from the current activation state is removed.

**Fig. 4:**
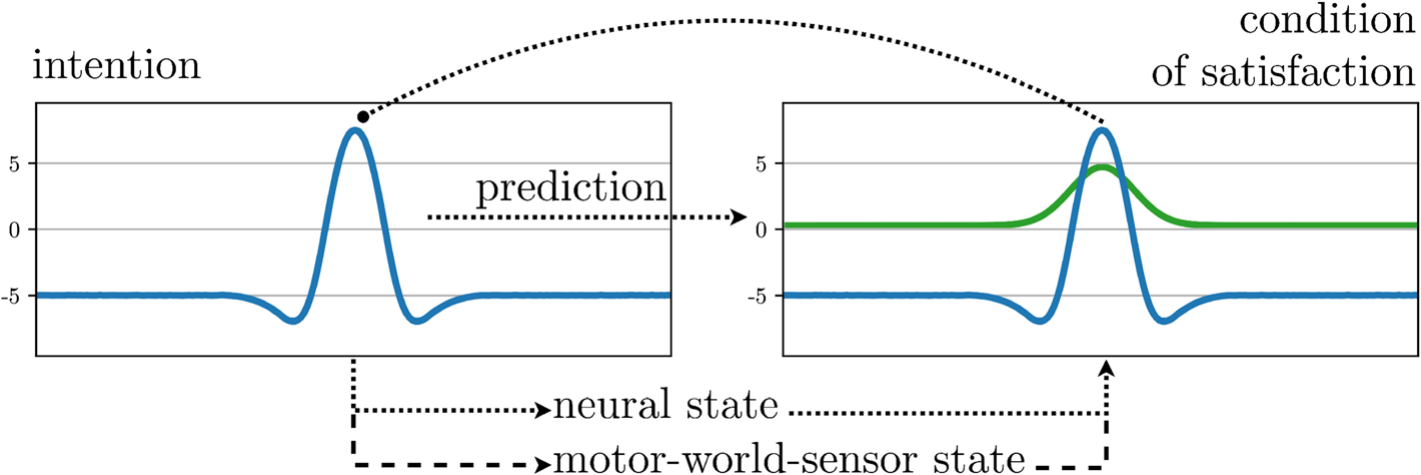
The coupling structure of the condition of satisfaction on which sequence generation is based in DFT. Arrows mark excitatory projections, the filled circle marks an inhibitory projection

The first element is organized in DFT through the concept of the *condition of satisfaction*, illustrated in Fig. 4. Inspired by the notion of intentionality in the philosophy of mind (Searle 1983), an *intentional state* is a neural activation pattern that drives whatever down-stream motor or cognitive acts are needed to achieve an intended outcome. This outcome is the condition of satisfaction, represented in a neural field which receives pre-activating input from the intentional state that predicts the outcome as well as internal or sensory input that reflects the outcome. When the field detects a match of predicted and observed outcome, it builds a peak in a detection instability. Its inhibitory projection onto the intentional field pushes that field through the reverse detection instability, deactivating the intentional state. As a result, the pre-activating input to the condition of satisfaction field falls away, inducing another reverse detection instability. In effect, the neural representations of the intentional state and its condition of satisfaction are reset.

A new activation peak may now arise in the neural architecture. Which peak arises where in the architecture may depend on different factors including inputs and working memories. This may entail selection from a number of possible local inputs (as in the gradient based approach to serial order; Henson and Burgess (1997)). Selection may be biased by directional coupling that implements what is known as “chaining” or “positional encoding” of serial order (Henson and Burgess 1997).

### Coordinate transforms

**Fig. 5:**
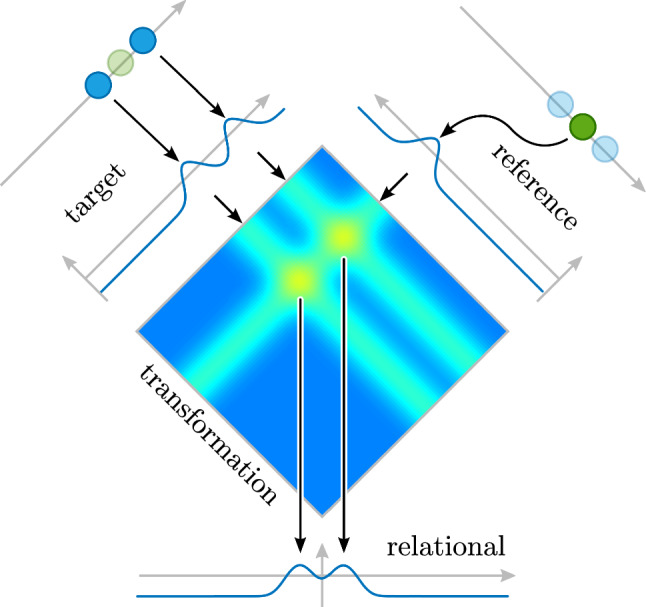
Steerable neural mapping to transform target objects into a coordinate frame centered on a reference object. The target field (upper left) contains peaks at the locations of two target objects (light blue). The reference field (upper right) contains a peak at the location of a reference object (green). The transformation (or gain) field is a joint representation of these two dimension. Either field provides sub-threshold ridge input to the transformation field. Peaks form where these ridges overlap. Projection from the transformation field along the diagonal creates a representation of the target objects centered on the location of the reference object (relational field). (Color figure online)

Coordinate transforms play an important role across many sensory-motor and cognitive tasks. The transformation from retinal to body-centered coordinates, for instance, lies at the core of visual cognition (for instance, Schneegans 2016). In neural networks, coordinate transforms amount to *steerable neural mappings*, projections from the original to the transformed representation that are steered by a parameter such as the direction of gaze relative to the body (Schneegans and Schöner 2012). Such mappings may be neurally implemented in what is known as gain fields (Pouget and Sejnowski 1997), essentially joint representations of the original and the steering dimension (Fig. 5). Peaks in the gain field form where input from the two sources overlaps. Any function of the two inputs to the gain field can then be computed by projecting out from the gain field to a transformed field using an appropriate pattern of connectivity. In the Figure, summing along the diagonal achieves the transformation to the desired coordinate frame.

## Grounding nested phrases

The representation and perceptual grounding of nested relational phrases, considered by some the backbone of grounded cognition (Barsalou 2008), is used in an exemplary case study to show how DFT may approach the cognitive competences idealized in the notions of productivity, compositionality, and systematicity. Building on earlier work (Lipinski et al. 2009, 2012; Richter et al. 2014, 2017; Kounatidou et al. 2018; Sabinasz et al. 2020; Richter et al. 2021; Sabinasz and Schöner 2022a, b), we first show how property and object concepts can be combined, then how spatial and movement relations can be grounded, and finally how conceptual structures can be represented and grounded.

### Perceptually grounding combined property and object concepts

**Fig. 6:**
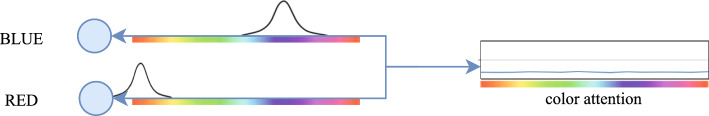
Color concepts are represented by neural nodes that are bi-directionally coupled to a color attention field. (Color figure online)

The grounding of simple property concepts, e.g. the color concept “red”, makes use of neural nodes that have bi-directional connections to a feature attention field that are patterned as a Gaussian centered on a prototypical feature value (Fig. 6). Thus, a feature concept may become activated by a peak in the feature attention field and, conversely, the activated feature concept may induce a peak in the feature attention field.

**Fig. 7:**
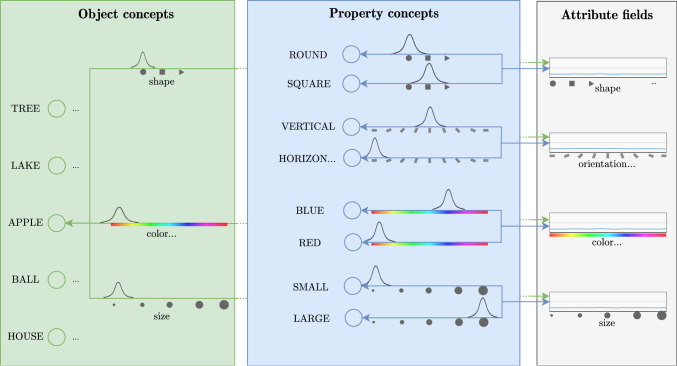
Neural dynamic representations of property and object concepts are linked to feature fields through connectivity patterns that encode the perceptual meanings of the concepts. Object concepts may project to multiple feature fields

Object concepts may directly project bi-directionally onto multiple such feature fields (Fig. 7) in a highly simplified neural dynamic implementation of prototype-based basic level concepts. This is sufficient for the perceptual grounding of object concepts, while the converse task of classifying an attended object would be expected to make use of more complex features as described in deep neural networks (Grieben and Schöner 2022).

**Fig. 8:**
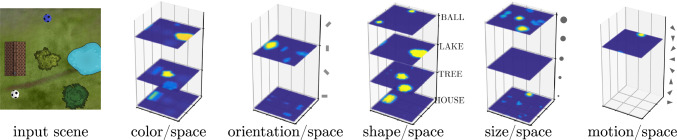
The visual scene (left) is represented by a set of feature/space perception fields. Localized activation peaks/blobs represent objects through their features/attributes. Adapted from Sabinasz and Schöner (2022b)

The perceptual grounding of property or object concepts consists of visually attending to an object in the visual array that matches the prototypical feature description. This makes use of *feature/space perception fields*, that each combine a representation of visual space with the representation of one or more feature dimensions (Fig. 8). Thus, supra-threshold activation localized at (*x*, *y*, *v*) represents an object at the location, (*x*, *y*), within the visual array that has feature value, *v*. Sharing the spatial dimensions across all feature/space fields, enables binding the different object properties across the different fields through space (Treisman and Gelade 1980; Schneegans et al. 2016).

**Fig. 9:**
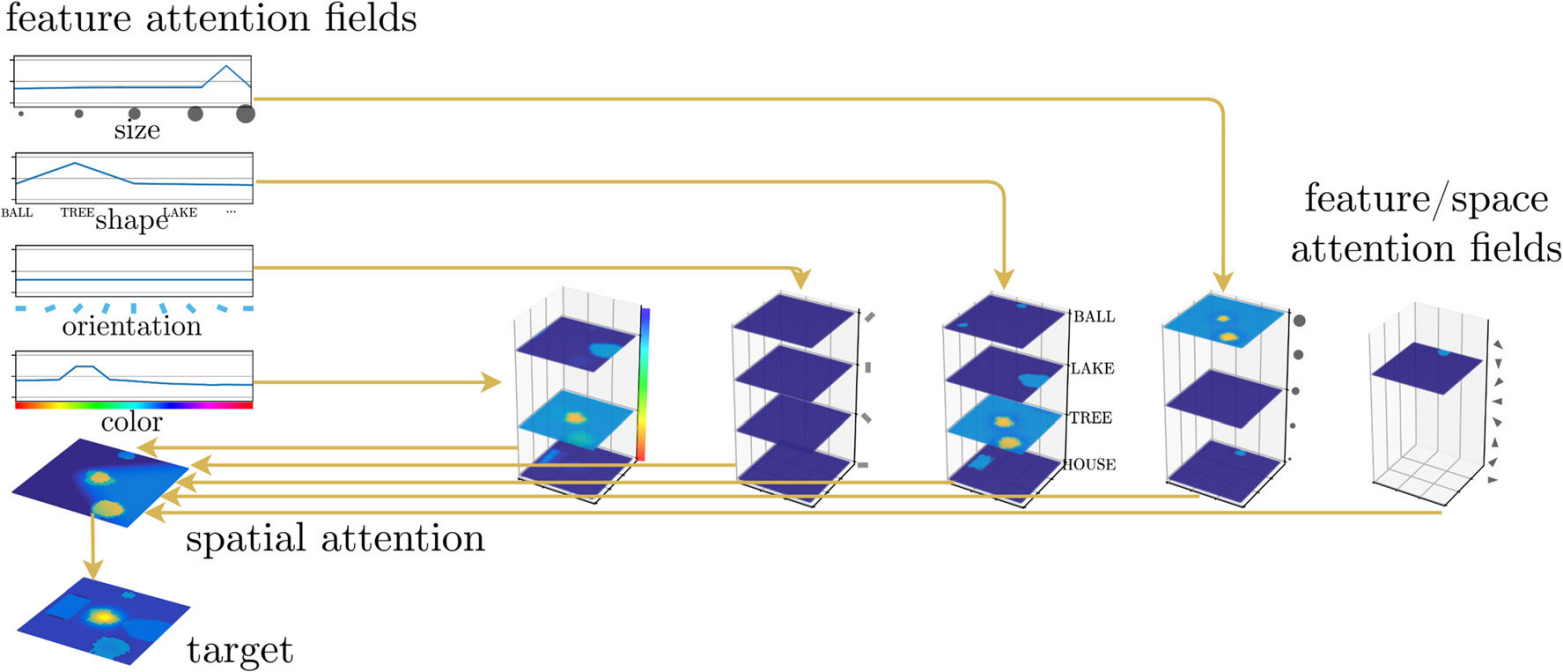
Feature/space attention fields receive input from the feature/space perception fields. Peaks in feature attention fields act as feature cues that boost activation in matching layers of the feature/space attention fields. Projection onto a spatial attention field induces peaks at locations where all features match. The target field selects a single target that matches the cued feature description. Adapted from Sabinasz and Schöner (2022b)

Selective attention to an object is represented by a supra-threshold activation peak in a target field that is driven from feature/space attention fields (Fig. 9). This selection results from *visual search* cued by a *feature attention field* for each feature dimension which is homogeneous across space and localized along the feature dimension (“slices” of input). The feature cues derive from activated property and object concept nodes. Summing along each feature dimension, these feature/space attention fields project onto a two-dimensional *spatial attention field*. Inhibitory inputs in proportion to the number of represented feature values (not shown) ensure that the spatial attention field only forms peaks on locations at which all cued feature dimensions match. The target field selects a single location among these, the outcome of the perceptual grounding process. A fuller account of visual search addresses how distractor objects are sequentially attended and discarded (Grieben et al. 2020), a complication neglected here.

### Perceptual grounding of spatial and movement relations

**Fig. 10:**
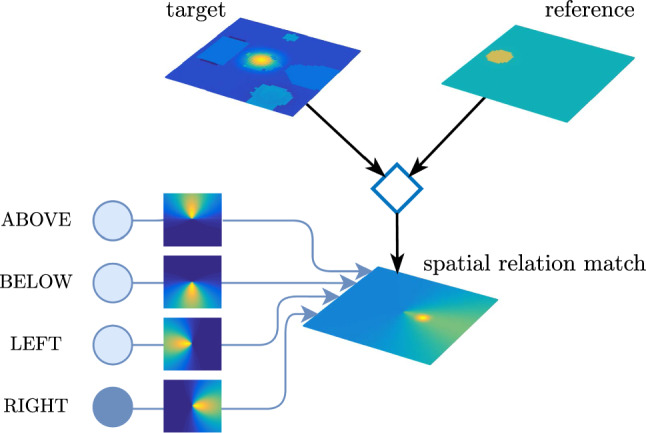
Spatial relation concept nodes (bottom left) are reciprocally coupled to a relation field through patterned connectivity that is illustrated using a color code for coupling strength. The relation field receives input from a spatial field representing target objects transformed through a gain field (diamond) into a coordinate frame centered on reference objects. Input to the target and reference field ultimately comes from the visual array (top) filtered by the spatial attention field

**Fig. 11:**
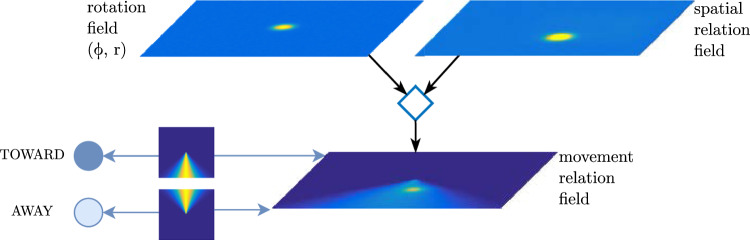
Movement relation concepts (left) are reciprocally coupled to a rotated relation field through patterned connectivity that is illustrated using a color code for coupling strength. The relation field is rotated (diamond) to align with the movement direction of target objects (rotation field). Adapted from Richter et al. (2021)

Spatial relation concepts are represented in DFT by neural nodes that are reciprocally coupled to a spatial relation field through patterned connectivity that encodes the spatial relation (Fig. 10). The spatial relation field represents target objects in a coordinate frame that is centered on reference objects through a coordinate transform. That field forms a peak if the relative spatial location of the target matches the active spatial relation concept. Movement relations are represented similarly (Fig. 11) based on a coordinate transform that rotates the relational field to align it to the movement direction of targets (Richter et al. 2021).

**Fig. 12:**
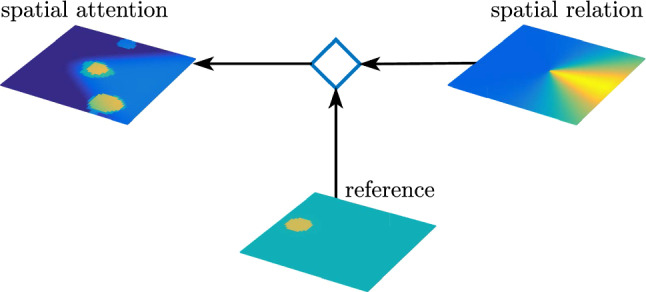
The spatial relation field is transformed (diamond) into a coordinate frame centered in the reference object and projects onto the spatial attention field

Relational concepts can also be used to directly guide visual search for target objects in the spatial attention field given a reference object and an activated relational concept (Sabinasz and Schöner (2022b); see also Grieben and Schöner (2022) for a similar mechanism in the context of guiding visual search based on relationships to anchor objects). This is based on a relation guidance field (Fig. 12) in which a spatial pattern encoding the prototype of a relation is coordinate transformed into a frame centered in a reference object and then projected onto the spatial attention field, effectively biasing attention towards objects that stand in the given spatial relation to the given reference object.

### Conceptual structure

**Fig. 13:**
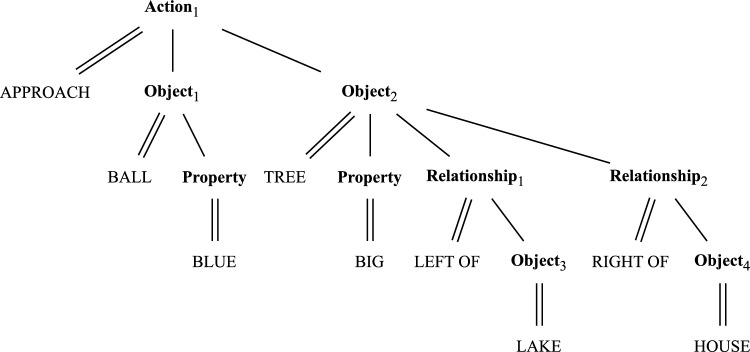
Exemplary conceptual structure for the sentence “the blue ball approaches the big tree, which is to the left of the lake and to the right of the house”. It encodes that there is an action characterized by the approach concept, being performed by an agent on a patient, where the agent is an object characterized by the ball concept that has a property characterized by the blue concept, and the patient is an object characterized by the tree concept that has a property characterized by the big concept, and stands in a relationship characterized by the left of concept to an object characterized by the lake concept, and in a relationship characterized by the right of concept to an object characterized by the house concept (this is slightly simplified from Jackendoff’s formalism)

Given neural mechanisms for grounding object or property concepts and simple relations between objects (e.g., “the tree is to the right of the house” or “the ball approaches the tree”), how would DFT combine multiple such conceptual units while expressing their interrelations, e.g., “the blue ball approaches the big tree which is to the left of the lake and to the right of the house”? We adopt the position that *conceptual structure* captures the way concepts are combined in a way that expresses their interrelations (Jackendoff 2002). In the example of Fig. 13, the concepts ball and blue are bound to the same object, representing the combined concept blue ball. The concepts tree and big are similarly bound to the same object, and that object stands in two spatial relationships to two further objects. The two bound objects blue ball and big tree are combined with the approach movement concept.

**Fig. 14:**
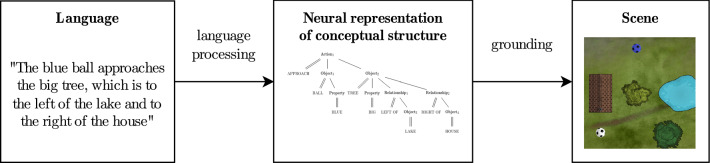
The neural representation of conceptual structure (center) is fed by language processing (left) not accounted for here, and guides the grounding (right)

What would be a possible neural representation of such conceptual structure in the language of DFT? Our hypothesis is that the conceptual structure is represented neurally as a working memory as the outcome of language processing (Fig. 14). Here we propose an account for this neural representation, but do not for the language processing that brings about that representation. Given the neural representation of conceptual structure, we then address how it may guide the perceptual grounding process of the objects in accordance with how they are arranged in the structure (see below).

A neural account of conceptual structure must address Jackendoff’s challenges (Jackendoff 2002; Sabinasz and Schöner 2022b). *The problem of 2* exemplified by the phrase “the small tree to the left of the big tree” requires that separate neural activation patterns represent the two trees. *The massiveness of the binding problem* exemplified by the phrase “the tree to the left of the lake which is to the left of the house” requires that a neural activation pattern encodes a single lake that is both the reference object of one relationship (“the tree to the left of the lake”) and the target object of another relationship (“the lake to the left of the house”). This requires flexibly binding an object to two different relationships in different relational roles.

**Fig. 15:**
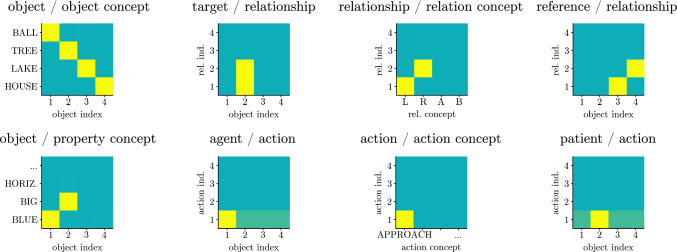
Neural field representation of the conceptual structure from Fig. 13. See text for details

Figure 15 illustrates the key idea of how such flexible interrelationships may be represented in DFT (Sabinasz and Schöner 2022b). Each concept node describing an object (object concept or property concept) is assumed to have an *index* dimension, so that it consists of a small number of copies (here four; see the object/object concept field).^1^ Similarly, each relation or action concept node comes in four copies, spanned by a relation or action index (see the relationship/relation concept field and the action/action concept field). These indices make it possible to express the interrelation between object, property and relation/action concepts as mentioned in a phrase. The same index is activated when the same object or relation is referenced. Different indices are activated when different objects or relations are referenced, even if these objects or relations are described using the same word. We assume this index resolution of the interrelations between objects and relations/actions comes from language processing, but do not account for exactly how that happens. Ultimately, the sequential processing of different object descriptions and relation/action descriptions would recruit new indices as needed (possibly using neural processes underlying sequence generation, see Sandamirskaya and Schöner (2010)). In effect, the indices enable flexible binding among concept nodes.

In Fig. 15, the *object/object concept field* binds “ball” to object index “1”, “tree” to object index “2”, and so on. The *object/property concept field* binds “blue” to object index “1”, and “big” to object index “2”. Thus, “ball” and “blue” are bound to one object index, and “tree” and “big” to another object index. The *relationship/relation concept field* binds “L” (for “left”) to relation index “1”, and “R” (for “right”) to relation index “2”. Roles of objects in relationships are then encoded entirely through the two types of indices in the *target/relationship* and *reference/relationship* fields. These encode, for example, that object 2 is in the target role of relationships 1 and 2 (encoding that the big tree is to the left of the lake and to the right of the house). Actions, their agents and patients, are treated analogously. For example, object 2 is the patient of action 1, encoding that the blue ball approaches object 2. This set of neural fields makes it possible to represent the different situations of Jackendoff’s problem of 2 and massive binding problem correctly. Note that the overall activation pattern in Fig. 15 encodes the conceptual structure from Fig. 13, but the same set of fields could encode any other conceptual structure as well.

### Perceptually grounding conceptual structure

**Fig. 16:**
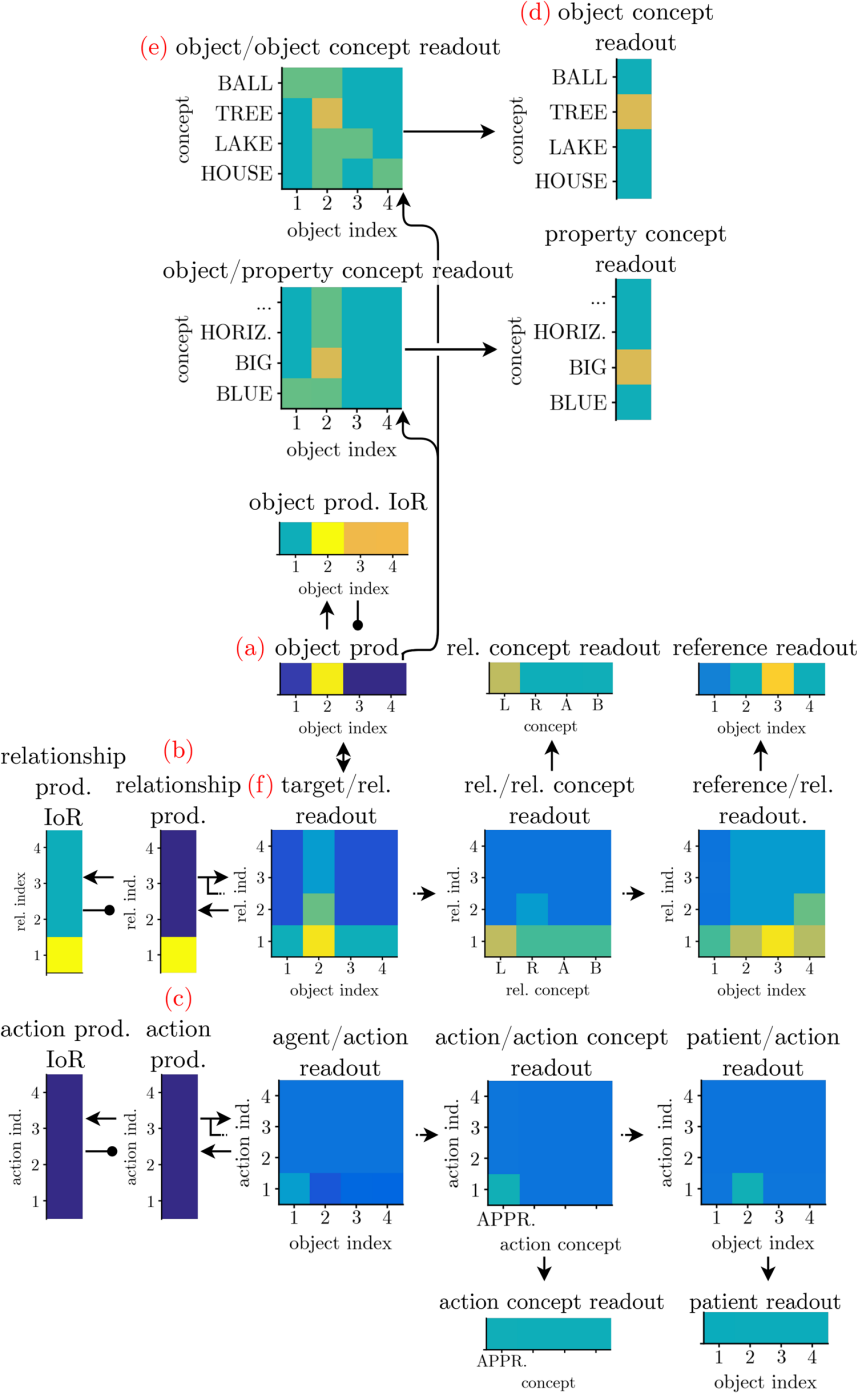
Interface between the conceptual structure and the grounding system to “read out” the currently selected object and relations/actions. Adapted from Sabinasz and Schöner (2022b)

Perceptually grounding a nested phrase represented in the conceptual structure is subject to the constraint that only one object can be attended at a time, and only one relationship or action description can be processed at a time. This constraint is inherent in the DFT approach to grounding (Schneegans et al. 2016), and consistent with empirical evidence (Logan 1994; Franconeri et al. 2012). The neural architecture illustrated in Fig. 16 provides for the neural substrate for such one-at-a-time processing: A selective *object production field* (a) defined over the object index dimension, a selective *relationship production field* (b) defined over the relationship index dimension, and a selective *action production field* (c) defined over the action index dimension. Entities of a given type compete for selection, controlled in each case by an inhibition-of-return (IoR) field.

To illustrate how a selected subset of objects/relations is projected out to the grounding system, we go through a few cases. First, consider the case when an object index was selected in the object production field (part (a) of Fig. 16). The concepts bound to that object index will project onto the scene representation to enable perceptual grounding. The object concept is read out via the *object/object concept readout field* (e), which receives subthreshold input from the object/object concept field and is boosted by ridge input at the selected index, effectively forming a peak where this ridge overlaps with the concept. This activates the current concept in the object concept readout field (d). An analogous mechanism enables reading out property concepts.

The relationships that contain the selected object as a target are read out through the *target/relationship readout field* (f) which receives input from the target/relationship field of the conceptual structure (not shown), and ridge input from the object production field (a). It projects onto the relationship production field (b), causing that field to select a relationship that contains the selected object in its target role. This enables that other information (relational concept and reference object index) bound to that relationship is also being read out through an analogous mechanism that culminates in a *relational concept readout field* and a *reference readout field*. When a relationship has been successfully grounded, the peak in the relationship field gets destabilized by the grounding system, enabling the selection of another relationship that has the currently selected object in its target role. When no such relationship exists anymore, the peak in the object production field gets destabilized, enabling the selection of a new object. Actions are handled analogously (c).

**Fig. 17:**
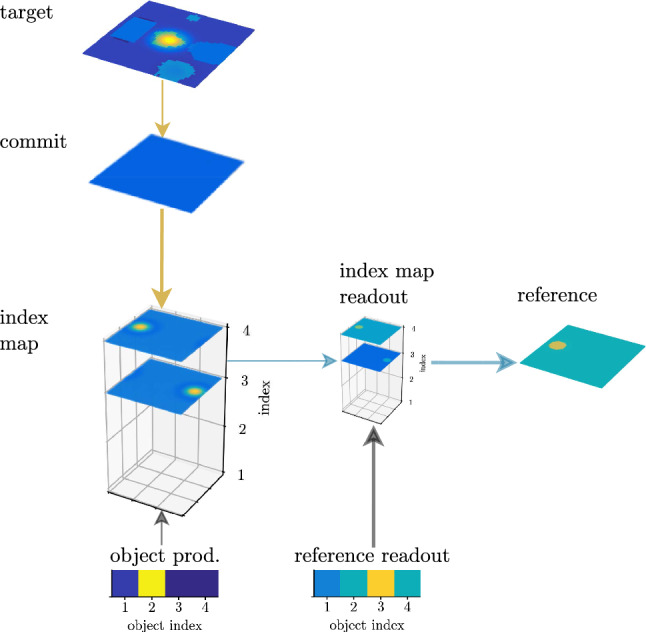
An index map may serve as a working memory of all the identified objects

**Fig. 18:**
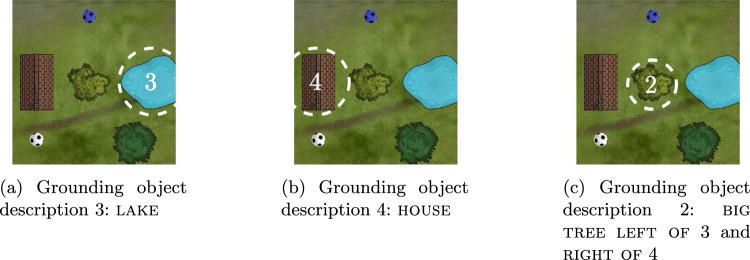
Grounding the phrase “the big tree which is to the left of the lake and to the right of the house” requires three grounding processes, where the possibility to ground the third (**c**) depends on having grounded the first (**a**) and the second (**b**) before, and having remembered their locations in a working memory

Once the target of an object description from the conceptual structure (characterized by a set of property concepts, object concepts, or relations) has been identified, its location must be memorized for future use during the perceptual grounding of the conceptual structure. This happens in a working memory field defined over the two spatial dimensions and the discrete object index dimension (Fig. 17). A peak in this index map at some location (*x*, *y*, *o*) represents that the object with index *o* has been found at location (*x*, *y*). Perceptual grounding occurs as a sequence of mental processing steps (to be explained below) lead to sequential entry of objects into this map. For example, while grounding the conceptual structure from Fig. 13, the map incorporates the locations of objects 3 and 4 with which object 2 stands in relations (Fig. 18a, b). This enables subsequent grounding of object 2.

An entry into the index map field occurs by combining the spatial input from the target field that represents the current target object with the currently activated object index (left column of Fig. 17). (The commit field plays a role in controlling the process.) To make use of an object already grounded in an earlier step, it is selected in the index map readout field based on input from the reference readout field.

### Complete neural dynamic architecture

**Fig. 19:**
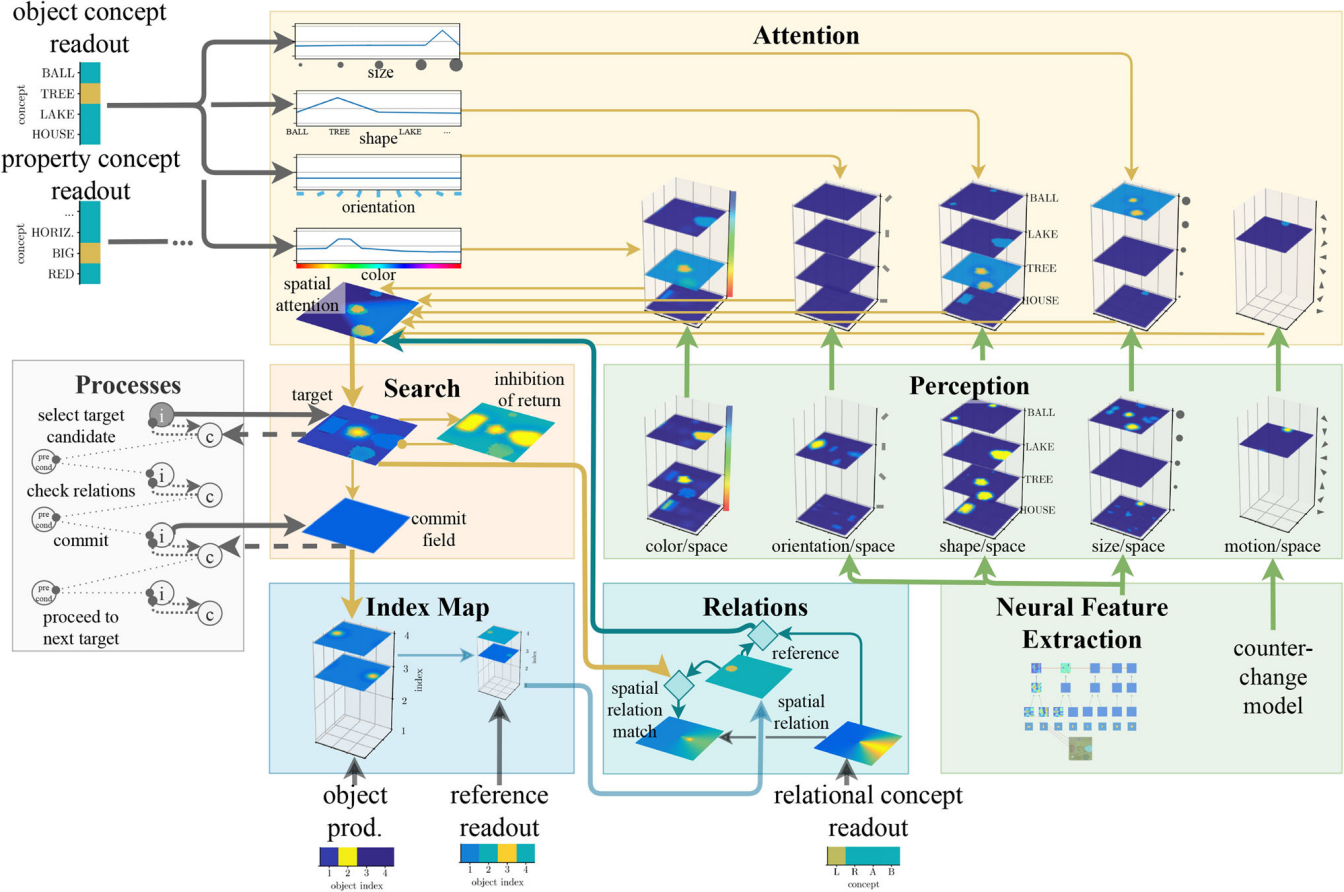
The model architecture for sentence verification. Adapted from Sabinasz and Schöner (2022b)

Only two components of the complete neural dynamic architecture (Fig. 19) for perceptually grounding nested phrases remain to be specified.^2^

First, feature input from the visual array to the architecture must be provided (bottom right in the Figure). This makes use of standard hierarchical forward neural networks for feature extraction and a neural mechanism for movement detection (the “counter-change” model, Berger et al. (2012)).

Second, the different components of the architecture need to be coordinated to autonomously generate the sequence of cognitive processing steps that lead to the grounding of the phrase (bottom left in the Figure). When an object index is selected for search in the object production field, four things have to happen in sequence: First, a candidate for the object has to be selected in the target field. Second, the relationships have to be checked. If one of the relationships does not match, the first step has to be repeated. An inhibition-of-return field biases selection to a different target candidate than before. Third, the object that matches all of the relationships has to be committed to the index map. Fourth, a new object index has to be selected in the object production field, and the first step starts again.

These four behaviors are implemented neurally making use of the DFT concepts for sequence generation. The *select target candidate process*, the *check relations process*, the *commit process*, and the *proceed to next target process* are each controlled through two neural nodes. The “intention node” represents that a process is active. Its connections to the rest of the architecture determine how the process achieves its predicted outcome. The “condition-of-satisfaction (CoS) node” represents that the process has successfully terminated. Connections from the architecture to the node determine the conditions under which this node becomes active. The serial organization of the processes is imposed through precondition nodes, which enable the activation of the next step only when the previous step has successfully terminated. More details about the individual processes are laid out in Sabinasz and Schöner (2022b).

**Fig. 20:**
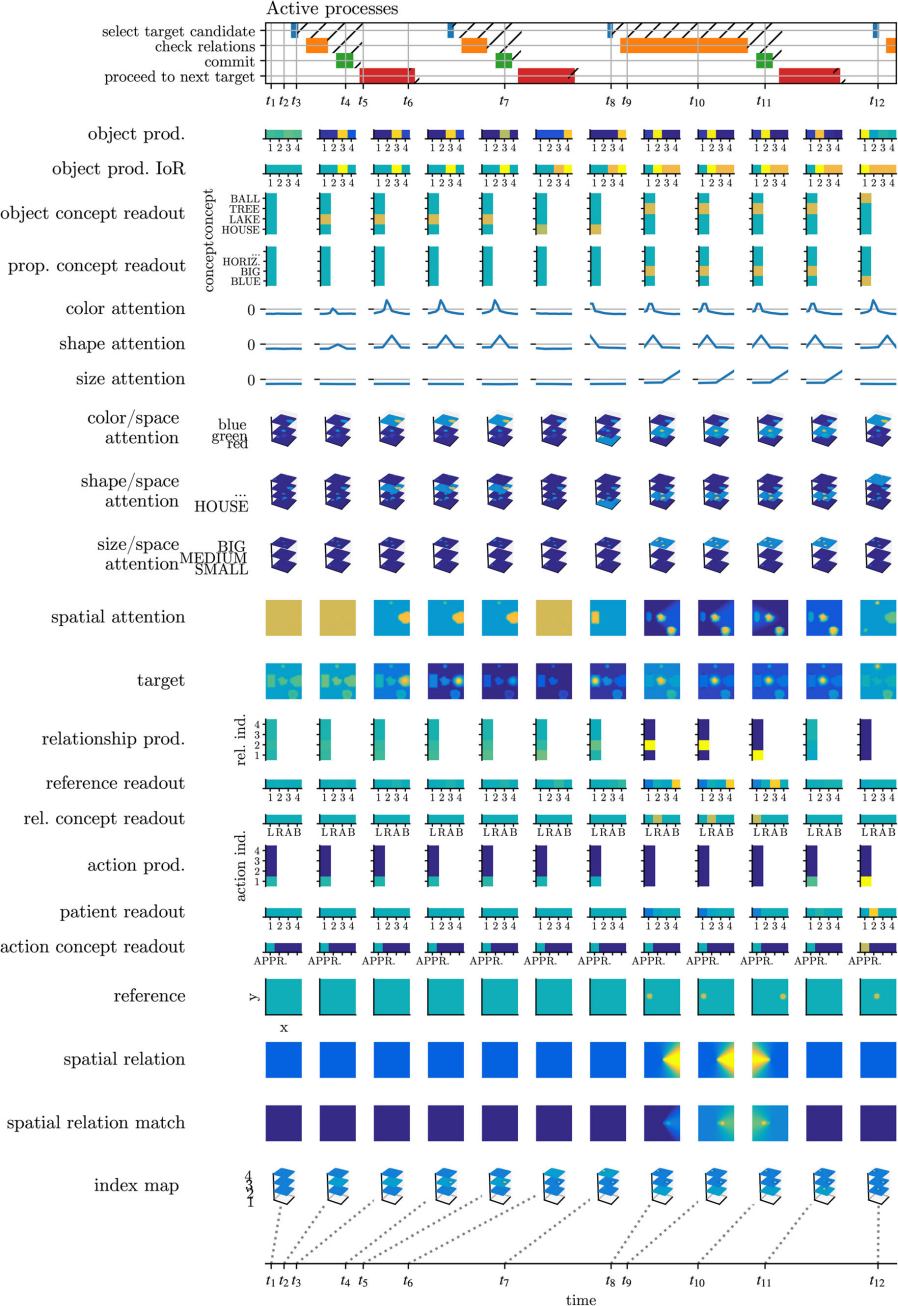
Activation snapshots of relevant fields as the architecture grounds the example phrase

Figure 20 shows a time course of activation through snapshots at discrete moments in time as the architecture grounds the sentence “the blue ball approaches the big tree, which is to the left of the lake and to the right of the house” in the scene shown in Fig. 18. Prior to the simulation, the conceptual structure fields have already been filled, leading to the activation pattern depicted in Fig. 15. Refer back to Fig. 13 for looking up the object indices and relationship indices assigned in this example phrase.

*Grounding of object 3 (the lake).* At time $t_2$, the object production field has selected object 3, reflecting a decision to search for that object (the lake). The readout mechanism has resulted in a peak on the lake concept in the object concept readout field. By time $t_3$, via the search mechanism, the target field has formed a peak on the spatial location of the lake in the target field. That peak reflects that a candidate for object index 3 is present at that location. It causes the CoS node of the select target candidate process to become active at $t_3$. At $t_4$, the commit process is active and has boosted the index map field, which in effect has formed a peak at the location of the target candidate and at index 3. That peak serves as a working memory of the location identified as the target of the object description with index 3. It activates the CoS node of the commit process. At $t_5$, the proceed to next target process is active and has provided transient inhibitory input to the object production field, destabilizing the peak. At $t_6$, a new peak has formed on object index 4, and the CoS node of the proceed to next target process has become active.

*Grounding of object 4 (the house).* Analogous mechanisms as before lead the system to ground object 4 (the house) and store it in the index map at $t_7$.

*Grounding of object 2 (the big tree to the left of 3 and to the right of 4).* By $t_8$, object 2 has been selected for grounding. The object concept readout field has formed a peak on the tree concept, which has highlighted the locations of trees in the spatial attention field. In addition, the property concept readout field has formed a peak on the big concept, which has highlighted the locations of big objects. In effect, the spatial attention field has high activation on big trees.

At the same time, the relationship production field has formed a peak on relationship 2. This has caused the reference readout field to form a peak on object index 4, which has provided ridge input into the index map readout field (not plotted), thereby causing a peak to form on the location of object 4, which has been transferred into the reference field. The peak in the relationship production field has also caused the relation concept readout field to form a peak on right of, which has caused the spatial relation field to form a pattern corresponding to the right of concept. That pattern has been transformed into a coordinate system centered on the reference object, effectively highlighting the area to the right of the reference object in the spatial attention field.

At $t_8$ a candidate for such an object is selected in the target field. Subsequently, the CoS node of the select target candidate process is activated, followed by the activation of the check relations process. At $t_9$, the already active relationship 2 is verified using the relational match detection mechanism, which compares the relative location of the target candidate in a coordinate system centered on the reference object to the activated spatial relation using the spatial relation match field. The formation of a peak in that field signals a match, which effectively results in the destabilization of the peak in the relationship production field and the formation of a new peak on relationship 1 by $t_{10}$. Analogous mechanisms as before lead the relational match detection mechanism to check whether the target candidate location is to the left of reference object 3. Since this is the case, the spatial relation match field again forms a peak, which results in the destabilization of the peak in the relationship production field. Since there are no further relationships that contain the currently active target object with index 2 in their target role, the relationship production field does not form a peak after $t_{10}$, which results in the activation of the CoS node of the check relations process. At $t_{11}$, the location of the target candidate is thus committed to the index map.

**Verifying the sentence (the blue ball approaches 2).** At $t_{12}$, analogous mechanisms as before have caused the reference field to form a peak on the location of object 2, and have caused the selection of a target candidate for the blue ball. Subsequently, the relational match detection mechanism may verify whether the approach relation holds between the target candidate and object 2 (as in Fig. 11).

The datasets generated during and/or analysed during the current study are not publicly available but are available from the corresponding authors on reasonable request.

## Discussion

We have outlined a neural theory of higher cognition that is based on a small set of key principles: (1) Cognition is based on localist representations that are endowed with dynamic stability by recurrent connectivity and that are grounded through their feed-forward connectivity from sensory and to motor surfaces. (2) Sequences of neural processing steps are generated autonomously through dynamic instabilities of the localist representations. (3) The neural operators implementing relations and actions can be generalized across space through coordinate transforms realized by steerable neural maps. (4) By binding the neural nodes through shared index dimensions, interdepencies among concepts within nested relational and action phrases can be neurally represented.

One might first think that localist representations do not scale well when the number and complexity of feature dimensions are increased (LeCun et al. 2015). Binding multiple localist representations through a shared dimension dramatically improves this scaling behavior. The capacity to dynamically control the creation of activation peaks makes this form of binding effective as peaks can be induced by input that is spatially homogeneous along some of the encoded dimensions. This is also how dynamic neural fields enable steerable neural maps: Ridges or slices of input effectively select one out of a continuum of possible mappings from one space to another. The coordinate transforms that are thus implemented endow neural networks with the power of function calls in that a neural circuit that implements a particular cognitive operation can be brought to bear on remote input.

Do these key principles enable a neural dynamic account for productivity, systematicity, and compositionality? In the exemplary case study we provided, productivity means that new phrases with new combinations of concepts and new dependencies among the concepts can be neurally represented and perceptually grounded. The binding index dimension, shared across all concepts, makes this possible. At the same time, that dimension points to inherent limits that might reflect actual limitations of human cognition. The entire conceptual structure operates as a working memory subject to capacity limits (Simmering and Perone 2012). The model set the depth of the index dimension to four to align with work on visual working memory (Luck and Vogel 1997), but realistic capacities may be larger, although not by much. Because new contents can be linked into the conceptual structure as grounding (or thinking) unfolds, this does not limit productivity per se, but it limits the depth of dependencies among phrases. And that limit may be psychophysically real.

The conceptual structure also imposes systematicity: the way in which dependencies among elements of sequences of phrases are organized is fixed by the dynamical structure of the representation. We made a proposal for the limited scenario we treated here. So the claim is not that this is the definite set of dynamic rules that govern conceptual structure. But the structure implies rules and that is the source of systematicity. Interestingly, the constraints on how the conceptual structure can be organized largely come from the requirements of grounding phrases. So in a certain sense, we could think of systematicity as arising ultimately from the sensory-motor grounding of cognition. Finally, the capacity to express relationships among elements of phrases and across phrases provides some form of compositionality: Grounding an element of a phrase may be based on the outcome of grounding another element of a phrase. So overall, a phrase is grounded by grounding its components in accordance with how these are arranged.

### Comparison to related theoretical approaches

Identifying the neural basis of higher cognition is a longstanding and broad challenge, with room for considerable debate, so we must be selective in discussing the relation of our proposal to the literature. We focus on neural process accounts and organize the discussion around the four key principles summarized above.

The *LISA* architecture (Hummel and Holyoak 2003; Doumas and Hummel 2012) is perhaps conceptually closest to our approach in that it too invokes localist representations of conceptual structure and of the associated semantic features (see also Doumas et al. (2008, 2022)). Structure is represented through a hierarchy of neural populations. At one layer of the hierarchy, neural populations encode symbols like bill, and at a lower level of the hierarchy symbols like male, adult, or human. Symbols may also include roles like lover defined in terms of symbols like has-emotion or emotion-positive. The connections between symbols at different levels encode the semantics of the symbols. Role-filler bindings like bill+lover are represented by neural populations that are connected to the symbols for the role and the filler, bill and lover. Finally, propositions like “Bill loves Mary” are represented by a neural population that has connections with the populations for role-filler bindings bill+lover and mary+beloved. Nested propositions like “Tom knows that Bill loves Mary” are encoded by neural populations with connections to populations for component role-filler bindings and propositions, and so on.

The notion of “binding” in LISA and related models differs from the notion as used in DFT. In DFT, we would speak about a joint representation of the role and the filler, so that a neuron would be tuned to both bill and lover. This is analogous to how activation within a feature/space field is tuned to both the feature and the spatial dimensions. The DFT notion of binding through space or index refers, instead, to a unique binding dimension shared across all neurons. For instance, if small neuronal populations tuned to bill or to lover were also tuned to an index dimension, the binding of bill to lover would be represented by activation for both populations being localized in the same place along the index dimension. This difference in how binding is achieved has important implications for how the representations scale with the number of concepts and combinations. In DFT, all “bindable” concept neurons must have that added dimension, which multiplies the size of the neural population by a constant factor (four in the model presented here). This enables representation of possible combinations. In LISA and related models, the number of units scales combinatorially with all possible bindings. As a consequence, the neural machinery that represents conceptual structure in DFT involves only a small set of populations tuned entirely to index dimensions. In LISA and related models, that machinery involves connections to any possible concept node, implying much more specific connectivity throughout the population of concept nodes (a criticism articulated earlier in Eliasmith (2013), Chapter 9).

A second point of contrast to LISA is that the actual sequence of processing steps within conceptual representations is not modelled neurally, but controlled by an algorithm. In that sense, LISA is not neurally autonomous as a process account. Finally, perceptual grounding in the sense of linking the conceptual representation to actual sensory inputs and performing attentional selection is not part of the LISA framework. The particular way DFT uses coordinate transforms to generalize neural circuitry required to perceptually ground relational and movement concepts is not, therefore, part of the LISA framework.

Vector-symbolic architectures (VSAs) (Smolensky 1990; Gayler 2023; Plate 1995; Levy and Gayler 2008) form a second major class of neural approaches to higher cognition. They are based on a quite different principle of neural representation, that is, in a sense, orthogonal to the assumptions of DFT. Here, vectors of neural activation built from the activation levels of a large set of neurons are the units of representation. Symbolic processing occurs by combining such vectors through binding and bundling operators that can take different mathematical forms (Schlegel et al. 2022). The original proposal for the binding operation (Smolensky 1990) expanded the dimensionality of the representation with each binding step, essentially by creating a joint representation of the components (see above). A critical innovation was the compression of these bound representations to the same dimensionality as the component vectors (Plate 1995; Levy and Gayler 2008). Together with a “clean-up” operation, this made it possible to use the vectors as symbols that may be combined to arbitrary depth. While the original formulation could be applied to both localist and distributed representations (Smolensky 1990), this more powerful form of VSA requires high-dimensional distributed representations in which vector-symbols are close to orthogonal to each other.

VSAs of this kind do not address how neural activation is autonomously generated and grounded. In fact, the encoding and decoding of the vector-symbols is a separate issue addressed outside the VSA proper, so perceptual grounding in our sense is not included in this framework. To autonomously generate neural activation vectors of the required form would require connectivity specific to each vector (for instance, of the Hopfield kind). That is not compatible with the very notion of freely combining such vectors. So the processing within VSAs of this classical form is not neurally plausible nor autonomous.

The neural engineering framework (NEF; Eliasmith and Anderson 2003) provides an alternative route toward neural implementation of VSAs (Stewart and Eliasmith 2012; Gosmann and Eliasmith 2019). In NEF, populations of integrate-and-fire neurons are tuned to represent the vector symbols. The vectors returned by binding or bundling operations are represented by new populations. Any given VSA can be mapped onto a network of such populations. The connectivity in that network is determined to ensure that the encoded vectors are “handed down” as symbolic operations are performed. SPAUN is a collection of models of higher cognition implemented in NEF (Eliasmith 2013; Choo 2018) which could be viewed as alternatives to the DFT framework laid out here.

NEF is theoretically neutral in that it could implement any neural network model in the form of integrate and fire neurons. Thus, NEF can also be used to implement DFT models (Turon et al. 2020). NEF may, in principle, link to sensory and motor representations (Eliasmith 2013). NEF/SPAUN could thus provide autonomous neural processing and a route toward perceptual grounding building on distributed representations. We argue, however, that NEF/SPAUN is not compatible with neural principles. In particular, the connectivity required to preserve the vector symbols violates the locality principle. This is because connections anywhere within a NEF implementation of a VSA must be “informed” by what vectors were originally encoded in the architecture. In summary, the NEF variant of VSAs remains orthogonal to the DFT based approach to higher cognition.

In fact, the spirit of NEF and VSA may be closer to the other fundamental approach toward a neural theory of higher cognition through something like a neural Turing machine. In that other perspective, neural mechanisms are sought that implement the abstract computational functions required to achieve symbol manipulation irrespective of how the symbols are linked to the sensory-motor domains. There is work along that line that literally uses the same mathematics as DFT does (beim Graben et al. 2008). Here, neural fields are used to neurally implement some of the machinery of VSAs in order to represent and parse nested phrases. Perceptual grounding and the linkage to the sensory-motor domain are left as separate issues. Thus, the dynamic properties of dynamic fields, including stability, do not play the same role in this account as it does in DFT. Sequence generation (beim Graben and Potthast 2014) is not based on release from stability. More recent work has impressively scaled the reach of these methods and begun to include ideas about sensory-motor grounding (Carmantini et al. 2017). Perhaps a path of convergence between these two different routes toward a neural theory of higher cognition is possible.

### Scope of the DFT framework for higher cognition

The DFT framework connects many different processes that may contribute to higher cognition. Many of the processing components have been tested against both neurophysiological and psychophysical data across different domains (as reviewed in Schöner and Spencer (2015)). For the specific architecture presented here, visual search (Grieben et al. 2020), and visuo-spatial working memory (Johnson et al. 2014) are empirically grounded components. The neural dynamics for the perceptual grounding of relations has been directly compared to human rating data earlier (Lipinski et al. 2012). Experimental signatures of the postulated mechanisms of grounding were uncovered in an experimental study using the mouse tracking paradigm (Lins and Schöner 2019). The mental map at the core of the neural machinery for grounding conceptual structure has been used earlier (Kounatidou et al. 2018) to account for experimental observations on spatial reasoning (Ragni and Knauff 2013). Somewhat more indirect support for the ordinal index system as a neural dimension comes from the observation of neurons in prefrontal cortex that are tuned to spatial location and ordinal rank (Xie et al. 2022).

Given the lack of quantitative psychophysics for the grounding of nested phrases, assessment of the account may be best framed in theoretical arguments. We illustrated that the model solves the “problem of 2” and the “massive binding problem” (Jackendoff 2002), for instance, and provides an explanation for role-filler independence in structured representations (Martin and Doumas 2020). The mechanism for grounding nested phrases was argued in an earlier variant of the model Sabinasz and Schöner (2022b) to be qualitatively in agreement with attentional studies during spoken language comprehension, as well as grammaticality judgment and eye-tracking studies during sentence parsing. The present model goes beyond this earlier version by including additional grammatical constructions (adjective-noun combinations, sentences with a verb) and extending the vocabulary that can be grounded.

### Scaling

Clearly, the neural architecture presented here only provides first steps toward higher cognition. How would the neural dynamic principles of conceptual structure scale as number and variety of concepts that must be linked into the neural machinery increases? Perceptual grounding by itself not a critical issue as the number of relevant feature dimensions is expected to by quite limited (DiCarlo and Cox 2007). The number of concepts, estimated in the hundreds of thousands (Brysbaert et al. 2016), is not a principle problem even for the postulated localist representations as these estimated numbers of concepts do not tax the neural resources of cortex. The capacity for composition boosts the reach of this form of representation.

The key potential bottleneck for this form of neural theory is the requirement that neural projections, realized by appropriate synaptic connectivity, would be extensive enough to link concepts into the conceptual structure. Recall that such connectivity must preserve the index dimension. Clearly, hundreds of thousands of neural population cannot be consistently connected in this way. One possibility is that multiple instances of a neural representation of conceptual structure exist, each linked only to a subset of concept nodes. These might be organized in semantic domains, with interesting implications for expressing dependencies among semantically very remote items. Perhaps analogical structure mapping may overcome some such limitations (Hesse et al. 2022).

### Learning

Although we have not addressed learning in this paper, DFT models are open to learning from experience (Part 3 of Schöner and Spencer 2015). Because peaks are largely generated by recurrent interaction, their instantiation in the detection instability may, in effect, amplify small differences in input or resting state. This fact lowers the demands on learning processes which only need to induce enough bias to nudge selection toward particular patterns. This mechanism has been used in DFT models to account for the effects of prior experience (Thelen 2001; Perone and Spencer 2013; Bhat et al. 2022).

Regular synaptic learning rules are a natural part of the DFT framework (Sandamirskaya 2014) and can be used to understand how the patterned connectivity arises that gives nodes their sensory-motor meaning (Sandamirskaya and Schöner 2010; Tekülve and Schöner 2020). When successful, such accounts explain how learning unfolds autonomously as a neural dynamic architecture generates mental and behavioral states.

## Appendix: Parameters

See Table 1.

**Table 1:** Relevant parameters of selected fields and nodes. The local excitation column contains the amplitude and the sigmas for each dimension in brackets. The lateral inhibition column contains the same for mid-range inhibition and global inhibition behind the slash. Default $\tau$: 100, default sigmoid $\beta$: 100

*Conceptual structure*			
Field	Resting level	Local excitation	Lateral inhibition
Obj. prod	$-8$	7	–
Obj. prod. IoR	$-5$	8	–
Obj./object conc. readout	$-5$	1	–
Obj./property conc. readout	$-5$	1	–
Object conc. readout	$-5$	1	–
Prop. conc. readout	$-5$	1	-
Relationship prod	$-$6.5	1	–
Relationship prod. IoR	$-5$	8	–
Reference readout	$-5$	5	–
Rel. conc. readout	$-5$	1	–
Target/rel. readout	$-12$	1	–
Reference./rel. readout	$-10$	1	–
Rel./rel. conc. readout	$-10$	1	–
Act. prod	$-$6.5	1	–
Act. prod. IoR	$-5$	8	–
Patient readout	$-5$	5	–
Act. conc. readout	$-5$	1	–
Agent/act. readout	$-12$	1	–
Patient/act. readout	$-10$	1	–
Act./act. conc. readout	$-10$	1	–
Act. conc. readout	$-5$	1	–
*Perception/Attention/relations*			
Color attention	$-$0.4	1. (3.)	0./$-$0.01
Shape attention	$-$0.4	1. (3.)	0./$-$0.01
Size attention	$-$0.4	1. (3)	0./$-$0.01
Color/space attention	$-5$	5. (2., 2., 1.)	0./0
Shape/space attention	$-5$	8. (2., 2., 0.1)	$-8.$ (4.,4.,0.2)/0
Size/space attention	$-5$	8. (2.,2., 0.1)	$-8.$ (4.,4.,0.2)/0
Spatial attention	$-5$	1. (3.)	0./0
Target	$-$6.3	30. (3.)	0./$-$0.14
Reference	$-5$	1. (3.)	0./0
Spatial relation	$-5$	1. (3., 3.)	0./0
Spatial relation match	$-$ 6.9	1. (3.,3.)	0./0
Index map	$-4$	21.(2., 2., 0.)	-18.(4.,4.,0.)/0
Index map readout	$-5$	1. (3.,3.,0.)	0./0
*Process organization*			
Select target cand. int	$-$4.5	1	–
Select target cand. CoS	-5	2	–
Check relations int	$-5$	1	–
Check relations CoS	$-5$	6	–
Check relation int	$-5$	1	–
Check relation CoS	$-5$	1	–
Proc. to next rel. int	$-5$	12	–
Proc. to next rel. CoS	$-5$	1	–
Commit int	$-5$	1	–
Commit CoS	$-5$	6	–
Proc. to next target int	$-5$	12	–
Proc. to next target CoS	$-5$	1	–

## Footnotes

1 To make the language uniform, we talk about index dimensions and fields although the index dimension is sampled discretely.

2 Relevant parameters of selected fields and nodes are contained in Table 1. The full set of parameters of the model is available as a human-readable JSON file at https://osf.io/s25ta. That file can be imported into the software cedar (Lomp et al. 2013) for simulating the model and reproducing simulation results.
